# Skewer: a fast and accurate adapter trimmer for next-generation sequencing paired-end reads

**DOI:** 10.1186/1471-2105-15-182

**Published:** 2014-06-12

**Authors:** Hongshan Jiang, Rong Lei, Shou-Wei Ding, Shuifang Zhu

**Affiliations:** 1 Institute of Plant Quarantine Research, Chinese Academy of Inspection and Quarantine, Huixinli 241, Beijing, 100029 China; 2 Department of Plant Pathology and Microbiology and Institute for Integrative Biology, University of California, Riverside, 900 University Ave, 92521 Riverside, USA

**Keywords:** Next generation sequencing, Adapter trimming, Approximate string matching, Local sequence alignment, Barcode demultiplexing

## Abstract

**Background:**

Adapter trimming is a prerequisite step for analyzing next-generation sequencing (NGS) data when the reads are longer than the target DNA/RNA fragments. Although typically used in small RNA sequencing, adapter trimming is also used widely in other applications, such as genome DNA sequencing and transcriptome RNA/cDNA sequencing, where fragments shorter than a read are sometimes obtained because of the limitations of NGS protocols. For the newly emerged Nextera long mate-pair (LMP) protocol, junction adapters are located in the middle of all properly constructed fragments; hence, adapter trimming is essential to gain the correct paired reads. However, our investigations have shown that few adapter trimming tools meet both efficiency and accuracy requirements simultaneously. The performances of these tools can be even worse for paired-end and/or mate-pair sequencing.

**Results:**

To improve the efficiency of adapter trimming, we devised a novel algorithm, the *bit-masked k-difference matching algorithm*, which has *O*(*kn*) expected time with *O*(*m*) space, where *k* is the maximum number of differences allowed, *n* is the read length, and *m* is the adapter length. This algorithm makes it possible to fully enumerate all candidates that meet a specified threshold, e.g. error ratio, within a short period of time. To improve the accuracy of this algorithm, we designed a simple and easy-to-explain statistical scoring scheme to evaluate candidates in the pattern matching step. We also devised scoring schemes to fully exploit the paired-end/mate-pair information when it is applicable. All these features have been implemented in an industry-standard tool named *Skewer* (https://sourceforge.net/projects/skewer). Experiments on simulated data, real data of small RNA sequencing, paired-end RNA sequencing, and Nextera LMP sequencing showed that *Skewer* outperforms all other similar tools that have the same utility. Further, *Skewer* is considerably faster than other tools that have comparative accuracies; namely, one times faster for single-end sequencing, more than 12 times faster for paired-end sequencing, and 49% faster for LMP sequencing.

**Conclusions:**

*Skewer* achieved as yet unmatched accuracies for adapter trimming with low time bound.

## Background

Adapter sequences are short oligonucleotides used to be ligated to the ends of DNA fragments of interest, so that they can be combined with primers for amplification. When the sequencing read length is greater than that of the target DNA, the adapter sequence is read out, sometimes partially, next to the unknown target DNA sequence. To recover the target DNA sequence, it is essential to identify the adapter sequence and trim it.

Adapter trimming was first used in small RNA (sRNA) sequencing, where typical lengths of the target fragments range from 18 nucleotides (nt) to 30 nt, while the typical read length is 36 nt. Another important application for adapter trimming is DNase-Seq, which is a high-resolution technique used to profile hypersensitive sites that are frequently bound by transcription factors. Recent studies showed that the sequencing of short reads (50–100 base pairs (bp)) gives better results [1].

For all the next-generation sequencing (NGS) applications including chromosomal DNA sequencing or complementary DNA (cDNA) sequencing, double-stranded DNA is first fragmented using nebulization or ultrasonics to obtain lengths of several hundreds bp. The ends of the fragments are then repaired and ligated with adapters. After purification, the adapter-ligated fragments are either poured on slides/chips as water-in-oils or attached to flow-cells for cluster generation. After several cycles of emulsion-PCR or bridge-PCR, the amplified templates are ready for sequencing. Fragmentation is a stochastic process that is influenced by the varied force field and thermodynamic stability of different parts of the sequence; e.g. parts with different GC content. To improve the enrichment rate and to cover the target genome or transcriptome more evenly, the sample preparation protocol usually requires fragments to be enriched in a specified length range. Even when longer average fragment lengths are chosen, the inherent nature of fragment size selection cannot always prevent short fragments and primer-dimmers from going on to the next stage. Therefore, there are nearly always reads that need to be trimmed. For the newly emerged Nextera long mate-pair (LMP) protocol, junction adapters that connect paired target fragments exist in all properly constructed fragments; thus, adapter trimming is essential to gain correct paired reads.

Adapter trimming is different from contaminant removal and is usually associated with NGS protocols where adapters are synthesized and specified by the reagent vendors. Given a known adapter pattern and a read sequence, adapter trimming is usually modeled as a semi-global sequence alignment, also called end-space free alignment, where any space at the end of or beginning of the alignment does not incur penalties.

Semi-global sequence alignment can be performed using the *Smith-Waterman* algorithm [2] with minor revisions of the boundary condition as implemented in *Cutadapt*[3], which has a time complexity of *O*(*mn*), where *m* is the pattern length, and *n* is the sequence length. A trickier solution, i.e. Ukkonen’s algorithm for *k*-difference matching [4], has an expected time of *O*(*kn*), where *k*<*m* is the maximum number of differences allowed. Further improvements in Ukkonen’s algorithm by bitwise parallelism were proposed by Myer [5] and implemented in Btrim [6], which has a time complexity of *O*(*mn*/*w*), where *w* is the word length of the computer; e.g. *w* equals 64 for a 64-bit machine. In practice, Myer’s bit-vector dynamic programming algorithm is the fastest *k*-difference matching algorithm currently available. However, Myer’s algorithm is more appropriate for searching patterns in text and is difficult to adapt to deal with sequencing quality values in NGS data.

Other popular adapter trimmers are available; e.g. *Fastx_clipper* in the *FastX-Toolkit* (http://hannonlab.cshl.edu/fastx_toolkit/), *SeqTrim* (https://github.com/dariogf/SeqtrimNext) [7], *TagCleaner*[8], and *EA-Tools* (http://code.google.com/p/ea-utils/). Recently published trimmers are: *SeqPrep* (https://github.com/jstjohn/SeqPrep) which focuses on paired-end (PE) reads; *Flexbar*[9], a flexible barcode demultiplexer that uses the *Needleman-Wunsch* algorithm [10] for pair-wise global sequence alignments, which has the same time complexity as that of *Smith-Waterman* algorithm; *Trimmomatic* (http://www.usadellab.org/cms/index.php?page=trimmomatic), which is a part of an integrated tool *RobiNA*[11]; *Scythe* (https://github.com/vsbuffalo/scythe), which uses a Naive Bayesian approach to classify contaminants in reads; *TrimGalore* (http://www.bioinformatics.babraham.ac.uk/projects/trim_galore/), which internally invokes *Cutadapt* with an extension to handle PE reads; *AdapterRemoval*[12], which is carefully tuned for trimming adapters from both single-end (SE) or PE reads; *AlienTrimmer*[13], which is based on *k*-mer decomposition for contaminant detection; and *NextClip*[14], which is dedicated to trimming adapters within Nextera LMP reads.

For sRNA sequencing data, a simple script can handle the adapter trimming task with acceptable accuracy and speed. However, trimming adapter sequences from genome sequencing data requires tools that are much more efficient, because the volume of data is much larger. Some very fast tools such as *Btrim* and *Trimmomatic* tend to over-simplify the underlying model. *Btrim* neglects the quality values associated with base calls for adapter trimming although it does use quality values for quality trimming [6]. *Trimmomatic* adopts a hash-based search followed by a simple score-based search, both of which neglect insertions and deletions for adapter matching (http://www.usadellab.org/cms/index.php?page=trimmomatic). To gain sufficient accuracy, most of the adaptor trimming algorithms use conventional pair-wise alignment algorithms such as the *Smith-Waterman* or *Needleman-Wunsch* algorithms; however, these alignment algorithms are inefficient for adapter trimming compared with more sophisticated algorithms [5].

In a typical application of adapter trimming, e.g. for sRNA sequencing, usually only a short prefix of the full-length adapter is scanned to reduce run time. However, this strategy may increase the possibility of random hits and cause biases in the trimmed sequences. Even when the specified pattern is of sufficient length, for reads where the 3’ end overlaps with a short prefix of the adaptor pattern, it is hard to judge whether the overlap is from finding the adapter sequence or from sequence homology. On the one hand, over-trimming of a bona fide part of the sequence causes loss of information; on the other hand, leaving an adapter untrimmed causes noise in the downstream analysis.

In this paper, we propose a *bit-masked k-difference matching* dynamic programming algorithm with *O*(*kn*) expected time and *O*(*m*) space in which the information within the adapter sequences is transferred into bits. We developed a carefully designed statistical scheme that incorporates quality values (and PE information when applicable), and implemented an industry-standard Linux program called *Skewer* to address the trimming problem accurately and efficiently.

## Results and discussions

### Features

The algorithm and statistical scoring schemes are implemented as a Linux program *Skewer* using C++. A comparison of the main features of *Skewer* with those of existing mainstream adapter trimmers are presented in Table 1.

**Table 1:** Main features of various adapter trimmers

	**Adapter trimming**						**Quality control**		**Other**			
**Method**	**5’**	**3’**	**SE**	**PE**	**LMP**	**Multi**	**Ns**	**Q**	**Barcode**	**Merge**	**gzip Files**	**MT**
FastX	×	○	○	×	×	×	○	×	○	×	×	×
SeqTrim	×	○	○	×	×	○	○	○	×	×	○	○
TagCleaner	○	○	○	×	×	×	×	×	×	×	×	×
EA-Tools	×	○	○	○	×	×	○	○	○	×	○	×
Cutadapt	○	○	○	○	×	○	×	○	×	×	○	×
TrimGalore	×	○	○	○	×	×	×	○	×	×	○	×
SeqPrep	×	○	×	○	×	×	×	×	×	○	×	×
Btrim	○	○	○	○	×	×	×	○	○	×	×	×
Scythe	×	○	○	×	×	×	×	×	×	×	○	×
Flexbar	○	○	○	○	×	○	○	○	○	×	○	○
Trimmomatic	×	○	○	○	×	○	×	○	×	×	○	○
AdapterRemoval	○	○	○	○	×	×	○	○	×	○	×	×
AlienTrimmer	○	○	○	○	×	○	×	○	×	×	×	×
NextClip	×	×	×	×	○	×	×	×	×	×	×	×
Skewer	○	○	○	○	○	○	○	○	○	×	○	○

For each method, the table shows if it is able to: i) identify adapters in the 5’ end of reads, ii) identify adapters in the 3’ end of reads, iii) process single-end (SE) reads, iv) process paired-end (PE) reads, v) process Nextera long mate-pair (LMP) reads, vi) search for multiple different adapters (Multi), vii) trim subsequences of multiple degenerative characters (Ns), viii) trim low-quality nucleotides (Q), ix) separate multiplexed reads based on barcodes, x) merge overlapped pairs into longer single-end reads, xi) process gzip files directly, and xii) run with multiple threads simultaneously (MT). (○: Yes; ×: No).

### Experiment environment

The server that was used for the experiments had 4 × 8-core Intel®; 2.67GHz CPUs, 1T memory, and RAID with bandwidths of 266MB/s and 262MB/s for reading and writing respectively. The operating system (OS) was the *Red Hat Enterprise Linux Server release 6.3*.

### Experiments on simulated data

#### General information

We simulated 10 million 100 bp + 100 bp PE Solexa reads from the *Arabidopsis thaliana* genome using ART, a NGS read simulator [15], with some revision on the source codes for simulating adapter-contaminated reads (http://sourceforge.net/projects/skewer/files/Simulator/). The trained profile was from the real sequencing data of *A. thaliana* where about 36% of the reads were contaminated with adapters. We compared *Skewer* with mainstream adapter trimmers that can handle PE reads as well as four representative adapter trimmers that can handle only SE reads.

To assess trimming quality, we defined the following metrics: *FP* (*false positive*) as the number of reads that were over-trimmed, either for trimming non-contaminant reads (*false trimming*), noted as *FP*_*ft*, or for *over-trimming* contaminant reads, noted as *FP*_*ot*; *FN* (*false negative*) as the number of reads that were under-trimmed, either for not trimming contaminant reads (*false retaining*), noted as *FN*_*fr*, or *under-trimming* contaminant reads, noted as *FN*_*ut*; and *TN* (*true negative*) as the number of untrimmed non-contaminant reads.

From these numbers, we defined the *positive predictive value* (PPV) as the ratio of the number of correctly trimmed reads to the number of trimmed reads; *sensitivity* (Sen) as the ratio of the number of correctly trimmed reads to the number of contaminant reads; and *specificity* (Spec) as the ratio of the number of untrimmed non-contaminant reads to the number of non-contaminant reads as follows:

(1) $$\begin{array}{ll} \text{PPV} & =\text{TP}/(\text{TP}+\text{FP}\_\text{ft}+\text{FP}\_\text{ot}+\text{FN}\_\text{ut})\; \end{array}$$

(2) $$\begin{array}{ll} \text{Sen} & =\text{TP}/(\text{TP}+\text{FN}\_\text{fr}+\text{FN}\_\text{ut}+\text{FP}\_\text{ot})\; \end{array}$$

(3) $$\begin{array}{ll} \text{Spec} & =\text{TN}/(\text{TN}+\text{FP}\_\text{ft})\; \end{array}$$

Finally, we defined the *Matthew’s correlation coefficient* (mCC), which is a quality measure for pattern recognition, as:

(4) $$\;\;\;\;\text{mCC}=\frac{\text{TP}\cdot \text{TN}-\text{FP}\cdot \text{FN}}{\sqrt{\left(\text{TP}+\text{FP})(\text{TP}+\text{FN})(\text{TN}+\text{FP})(\text{TN}+\text{FN}\right)}}$$

#### Primary result

Each method was run with its default parameters, except that the minimum output fragment length and the thread number (if applied) were set to 1.

The results obtained from these runs are listed in Table 2 and details are available in Table S1 of Additional file 1. *FastX*, an earlier and widely adopted NGS adapter trimmer, had a relatively low *mCC* (0.6683) and a low processing speed (0.92*Mbp*/*s*); *SeqTrim* had a similar overall performance as *FastX* (0.6618), but it had the slowest processing speed (0.03*Mbp*/*s*) of all the trimmers tested despite its extensive logging utility; *TagCleaner* was clearly the most conservative of the trimmers (*FP*=0), but it had the lowest sensitivity (45.50*%*) and was notably slower than *FastX* (58.7*%* of the speed); *EA-Tools* had the highest sensitivity (99.72*%*) for processing SE reads and was orders of magnitude faster (13*X*∼400*X*) than the slow trimmers; *Cutadapt*, the most widely accepted adapter trimmer, exhibited a good compromise between sensitivity and specificity (96.27*%* vs. 96.93*%*), and had the highest *mCC* (0.9286) among the existing tools for processing SE reads; *TrimGalore*, a wrapper for *Cutadapt*, had a performance that was equivalent to *EA-tools* with default settings, but it was considerably slower than *EA-tools* (28.2*%*∼31.6*%* of the speed); *SeqPrep*, a dedicated PE reads adapter trimmer and merger, had the highest *mCC* (0.9975) among the existing tools for processing PE reads, but it was slow (0.64*Mbp*/*s*); *Btrim* had the highest speed (23.63*Mbp*/*s*) for adapter trimming, but it had low sensitivity (53.44*%*); *Scythe* had an *mCC* similar to that of *Cutadapt* for SE reads adapter trimming, but was more conservative; *Flexbar* had slightly lower metrics and about 20% lower processing speed than *TrimGalore*; *Trimmomatic* was among the most conservative ones, but it had an acceptable sensitivity (72.31*%*) and a relatively high speed (16.73*Mbp*/*s*); *AlienTrimmer* had similar metrics to *Btrim*, but was much slower (1.64*Mbp*/*s*); and *AdapterRemoval* had a similar overall performance as *SeqPrep* for PE reads processing, but unlike *SeqPrep* it can also handle SE reads.

**Table 2:** Performance of adapter trimmers on 2Gbp simulated data

**Method (Single End/Paired End)**		**Speed (Mbp/s)**	**Memory (Mb)**	**PPV (%)**	**Sen. (%)**	**Spec. (%)**	**mCC**
FastX	SE	0.92	13.8	68.90	90.84	77.97	0.6683
SeqTrim	SE	0.03	115.7	67.07	85.27	81.24	0.6618
TagCleaner	SE	0.54	37.6	100.0	45.50	100.0	0.5898
EA-Tools	SE	12.04	17.7	59.24	99.72	61.32	0.6010
	PE	11.54	30.0	59.16	99.43	61.36	0.5983
Cutadapt	SE	4.36	34.5	94.55	96.27	96.93	0.9286
	PE	3.44	42.8	94.55	96.00	96.93	0.9266
TrimGalore	SE	3.81	19.4	59.24	99.72	61.32	0.6010
	PE	3.26	19.6	59.16	99.44	61.36	0.5984
SeqPrep	PE	0.64	22.0	99.84	99.82	99.92	0.9975
Btrim	SE	23.63	11.2	99.96	53.44	100.0	0.6503
	PE	5.79	15.3	99.89	53.30	100.0	0.6490
Scythe	SE	3.15	11.2	99.56	90.86	99.92	0.9283
Flexbar	SE	2.82	9.5	57.90	99.12	59.48	0.5814
	PE	2.70	9.7	57.77	99.09	59.29	0.5795
Trimmomatic	SE	16.73	2593.0	99.99	72.31	100.0	0.7907
	PE	16.40	2292.0	100.0	71.54	100.0	0.7850
AdapterRemoval	SE	1.67	6.3	75.09	97.74	81.89	0.7675
	PE	0.73	8.3	99.93	94.47	99.97	0.9566
AlienTrimmer	SE	1.64	2319.9	85.62	57.11	99.96	0.6769
	PE	1.61	2248.9	83.71	55.67	99.95	0.6659
Skewer	SE	8.79	13.6	94.56	96.32	96.93	0.9291
	PE	8.88	22.2	100.0	99.86	100.0	0.9989

Methods that process only single-end (SE) or paired-end (PE) reads are indicated.

The results of the runs listed in Table 2 show that *Skewer* outperformed all the mainstream tools in terms of *mCC* for both SE and PE trimming (0.9291 and 0.9989 respectively), although *Skewer* was only marginally better than *Cutadapt* and *Scythe* in SE trimming. Furthermore, *Skewer* was substantially faster (one times faster for SE and more than 12 times faster for PE trimming) than the tools that had comparative performances.

*Trimmomatic* and *AlienTrimmer* both used above 2*G* bytes peak memory. Most of the other trimmers used less than 50*M* bytes memory except *SeqTrim*, which used 115.7*M* bytes. Although *Skewer* did not have the least memory usage, its memory consumption (less than 35*M* bytes, see Table S2 of Additional file 1 for details) was far from a bottleneck on a 64-bit computer. In fact, *Skewer* uses additional memory to facilitate the processing of IUPAC (International Union of Pure and Applied Chemistry) characters and for parallel computing.

#### Scalability for parallel computing

We ran various adapter trimmers that support multi-threading to compare their scalability under a parallel computing environment. *SeqTrim* was excluded because it is too slow to gain comparative speed even with tens of threads. In addition, *Cutadapt*, *AdapterRemoval*, and other tools were not included because they currently lack a multi-threading function. In the eight threads case, for both the uncompressed and compressed inputs, *Skewer* achieved the highest speedup among the adapter trimmers tested (7.87 for uncompressed input, and 4.54 for compressed input) (see Table S2 of Additional file 1 for details).

#### Receiver operating characteristic (ROC) curves

**Figure 1:**
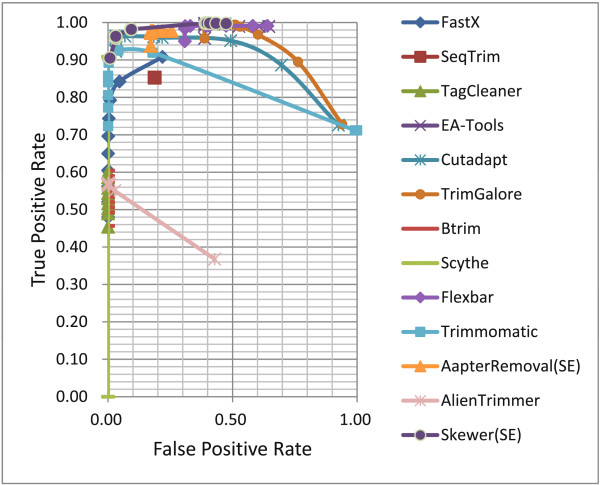
**ROC curves of various adapter trimmers for processing single-end reads of simulated data.** ROC: receiver operating characteristic.

**Figure 2:**
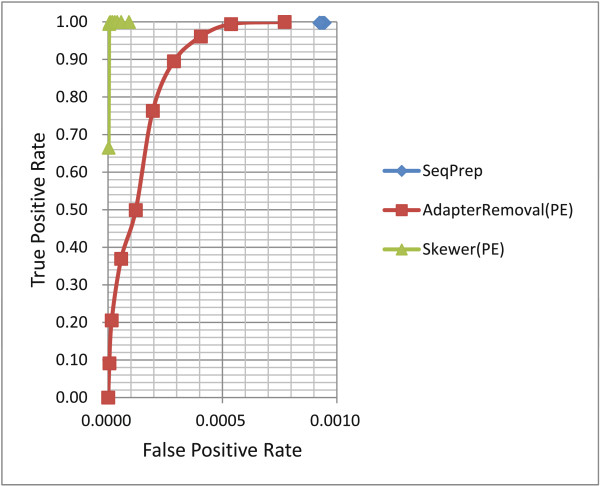
**ROC curves of various adapter trimmers for processing paired-end reads of simulated data.** ROC: receiver operating characteristic.

ROC curves for various adapter trimmers under different stringencies were plotted and are shown in Figure 1 and Figure 2 (see Table S3 of Additional file 1 for details).

In high stringency (left) regions, both *Trimmomatic* and *Cutadapt* performed well in that they had low FPRs (false positive rates) and high TPRs (true positive rates); however, as the stringency decreases, both their performance degrade gradually (Figure 1). A similar trend was seen for *TrimGalore* where the ROC curve shifted to the upper-right region. This implies that these trimmers greedily picked up the first candidate that met the stringency rather than select the optimal one. The ROC curve for *AlienTrimmer* was similar to the above ones, but with a worse performance. *FastX* may adopt some optimization technique, however, its performance was worse than those of *Trimmomatic* and *Cutadapt* within all the stringency range. Other adapter trimmers showed advantages on a specific metric; e.g. *AdapterRemoval*, *Flexbar*, and *EA-Tools* were the most sensitive, while *TagCleaner*, *Btrim*, and *Scythe* were the most conservative. *SeqTrim* appeared only as a dot in the ROC curves plot, because it does not provide a stringency threshold. *Skewer* outperformed the other adapter trimmers in that it had the least FPR to gain a specific TPR, when TPR > 95%.

ROC curves for the adapter trimmers that are aware of PE information were plotted and are shown in Figure 2. Other trimmers that can process PE reads have worse ROC curves than corresponding ROC curves for processing SE reads since the second reads usually have lower sequencing qualities. From Figure 2, we can see that *Skewer* had a nearly perfect ROC curve close to the upper-left corner. For example, it achieved a TPR of 99.951% with a FPR of 0.001%.

### Experiments on real data

#### sRNA sequencing data for *Caenorhabditis elegans*

A recently published real sRNA data set (short read archive [SRA:SRR014966]) [16], which includes 14,251,981 reads of small non-coding RNA (ncRNA) from *C. elegans*, was used to evaluate the adapter trimmers. Because it is hard to recover all the underlying sRNA fragments for sequencing, we aligned the trimmed reads to the reference genome and used delta of the number of uniquely aligned reads relative to the number of uniquely aligned raw reads, noted as *TT* (*true trimming*), as a substitute for *true positive*. We also used delta of number of non-uniquely aligned reads relative to the number of non-uniquely aligned raw reads, noted as *FT* (*false trimming*), as a substitute for *false positive*. The rationale was that correct-trimming tends to change unaligned fragments to uniquely aligned fragments (*true positive*), while over-trimming tends to change uniquely aligned fragments to non-uniquely aligned fragments (*false positive*). Note that these metrics tolerate tiny mistakes that can be rescued by the alignment software and are useful for practical evaluation.

To evaluate the performances under various trimming stringencies, all the tools were used to trim adapter sequences from the *C. elegans* data set using various trimming stringency. Next the processed reads were aligned to the *C. elegans* genome [17] (version 10) using Bowtie2 [18] (version 2.1.0). We then used the above metrics for final plotting, with higher *FT* representing lower stringency.

**Figure 3:**
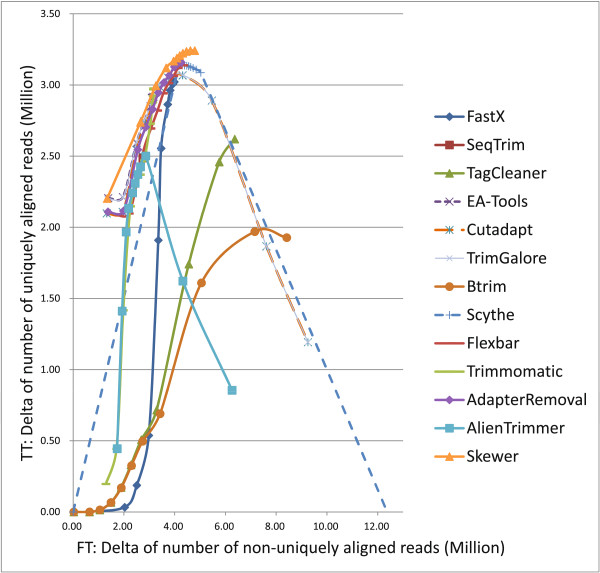
Performance of various adapter trimmers on real small RNA data [SRA:SRR014966].

The results are presented in Table S4 and Table S5 of Additional file 2, and illustrated in Figure 3. *AdapterRemoval* and *Flexbar* exhibited similar performance curves, while *AdapterRemoval* was slightly better than *Flexbar* within all the tested stringency range; and *TrimGalore* and *Cutadapt* had similar curves, while *TrimGalore* was slightly better than *Cutadapt* at all the stringencies. Under high stringency, *EA-Tools*, *Skewer*, and *TrimGalore* shared the first rank in terms of low *FT* and high *TT*; *Trimmomatic*, *AdapterRemoval*, and *Schythe* were ranked second under middle stringency, middle low stringency, and low stringency respectively; and *Skewer* ranked first at all the stringencies.

#### Paired-end RNA sequencing data for *Drosophila simulans*

A real RNA-Seq data set with 27,005,344 pairs of 101 bp reads (short read archive [SRA:SRR330569]) from the gonads and carcasses of *D. simulans* was used to compare the performances of the adapter trimmers in trimming artificial contaminants from PE reads.

For the evaluation, we first used each of the tools that can deal with PE reads with default setting to trim adapters from the reads, with the exception that the minimum output fragment length was set to 20 and quality trimming was inhibited. We then used TopHat [19,20] (version 2.0.10) to align the processed reads to the reference genome of *D. simulans*[21] (dsim revision 1.4). Finally the number of uniquely and concordantly aligned pairs was used as the performance metrics.

**Figure 4:**
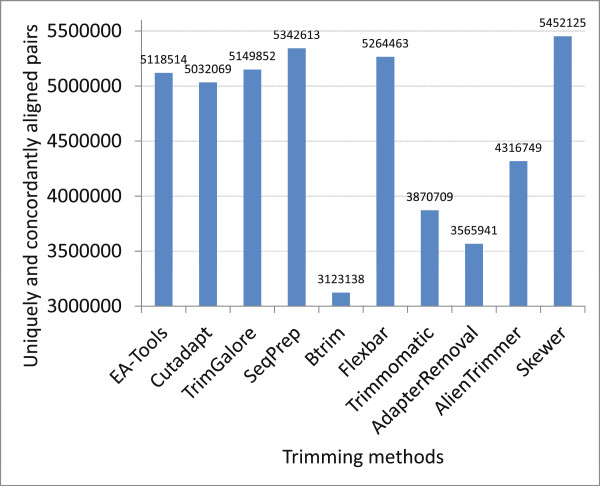
Performance of various adapter trimmers on real paired-end data [SRA:SRR330569].

The results are presented in Table S6 of Additional file 3 and illustrated in Figure 4. *Skewer* outperformed the other adapter trimmers in terms of the number of uniquely and concordantly aligned pairs of the trimmed PE reads. *Trimmomatic* and *AdapterRemoval*, both of which performed well in processing the sRNA data, performed poorly in processing the long PE data. This finding implies that these tools may be tuned specifically for trimming adapters from sRNA data. Similarly, *Btrim* also performed less well with the PE data in this experiment. After investigating the processed data, we found that *Btrim* could recognize only the occurrence of the whole adapter sequence with a limited tolerance for insertions and deletions. It should be noted that all quality trimming was inhibited from these experiments to compare the adapter trimming performance alone. However, in real applications, quality trimming, which is outside of the scope of this paper, has been reported to improve the mapping rate and facilitate downstream data analysis [22].

#### Nextera long mate pair (LMP) data for *Arabidopsis thaliana*

A 5-kb insert size Nextera LMP library of *A. thaliana* Col-0 with 6,602,426 pairs of 251bp reads (European Nucleotide Archive [ENA:ERA264981]) and a 400-bp insert size Illumina HiSeq PE library of the same species with 17,341,797 pairs of 100 bp reads [ENA:SRR519624] were sequenced previously and used to demonstrate the utility of NextClip [14], a dedicated tool for trimming adapters from Nextera LMP libraries.

To compare *Skewer* with NextClip, we followed a validation procedure similar to the one described for NextClip [14]. Briefly, the LMP library was first trimmed using the adaptor trimmer. Then the trimmed LMP reads and the PE reads were de novo assembled using ABySS [23]. The time needed for the adapter trimming and the N50 lengths of the scaffolds were used as the metrics for the evaluation.

The result is listed in Table 3 (see Additional file 4 for relevant commands in detail), from which we can see that Skewer marginally outperforms NextClip in terms of assembly statistics (N50 length etc.) of the trimmed reads. In addition, Skewer is about 49% faster than NextClip in single thread mode.

**Table 3:** Comparison of NextClip and Skewer in processing Nextera long mate-pair (LMP) reads (ERA264981)

**Reads for scaffolding**	**Time for trimming adapters from Nextera LMP library (s)**	**Assembly**		
		**Pieces**	**N50 length**	**Total length (Mbp)**
None	N/A	14097	19150	105.80
Paired end (PE) only	N/A	11781	23496	104.84
PE and NextClip processed	1480.39	6080	309342	111.55
PE and Skewer processed	993.49 (single thread)	5806	312317	112.52
	155.96 (8 threads)			

## Conclusions

We presented a novel algorithm and applied it to adapter trimming. The inherent advantage of the proposed *bit-masked k-difference matching* dynamic programming algorithm makes it possible to search adapter sequence pattern in an exhaustive yet efficient manner. Moreover, by using carefully designed scoring schemes for adapter pattern matching in both SE and PE sequencing data, the resultant *Skewer* tool was shown to achieve accuracies that were not matched by other similar tools that are currently available. Importantly, *Skewer* was not optimized for specific applications (e.g. sRNA sequencing); however, compared with other adapter trimmers, it performed well over all NGS applications.

Read lengths and throughputs of NGS technologies are likely to keep increasing; therefore, efficient and accurate adapter trimming methods will continue to be important in the preprocessing steps in applications such as genome resequencing, de novo sequencing, transcriptome sequencing, as well as sRNA sequencing.

## Methods

### Problem definition

#### Notation

The *Levenshtein distance* between two strings *A*=*a*_1_*a*_2_… *a*_|*A*|_, *B*=*b*_1_*b*_2_…*b*_|*B*|_, noted as ∥*A*,*B*∥_*l* *e* *v*_, is given by a recursive formula (assuming |*A*|>0,|*B*|>0):

$$\;{∥A,B∥}_{\text{lev}}\;:=\text{min}\;\left\{\;\;\;\begin{array}{l} {∥a_{1}a_{2}\ldots a_{|A|-1},b_{1}b_{2}\ldots b_{|B|-1}∥}_{\text{lev}}+\delta_{a_{\left|A\right|},b_{\left|B\right|}}\\ {∥a_{1}a_{2}\ldots a_{|A|-1},B∥}_{\text{lev}}+1\\ {∥A,b_{1}b_{2}\ldots b_{|B|-1}∥}_{\text{lev}}+1 \end{array}\right)$$

where *δ*_*a*,*b*_=1 if *a*≠*b*, otherwise *δ*_*a*,*b*_=0. When *min*(|*A*|,|*B*|)=0, ∥*A*,*B*∥_*l* *e* *v*_:=*max*(|*A*|,|*B*|).

#### *k*-difference problem

Given a sequence *S*=*s*_1_*s*_2_…*s*_*n*_, a query pattern *P*=*p*_1_*p*_2_…*p*_*m*_, and a threshold *k*(0≤*k*<*m*), search all substrings of *S*, noted as {*P*^′^}, such that ∥*P*^′^,*P*∥_*l* *e* *v*_≤*k*.

#### Extended *k*-difference problem

Given a sequence *S*=*s*_1_*s*_2_…*s*_*n*_, a query pattern *P*=*p*_1_*p*_2_…*p*_*m*_, and a threshold *e*(0≤*e*<1,⌊*n*×*e*⌋=*k*), search all substrings of *S*, noted as {*P*^′^}, such that ∥*P*^′^,*P*∥_*l* *e* *v*_≤*k*; and all suffixes of *S*, noted as {*S*^′^}, such that ∃ a prefix of *P*, noted as *P*^′^, and ∥*S*^′^,*P*^′^∥_*l* *e* *v*_≤|*S*^′^|×*e*

### Algorithms

For the *k-difference problem*, the classic approach [2] computes a (*m*+1)×(*n*+1) dynamic programming matrix C[0..m,0..n] using the following recurrence:

$$C[i,j]=\text{min}\left\{\begin{array}{l} C[i-1,j-1]+\delta_{\text{ij}}\\ C[i-1,j]+1\\ C[i,j-1]+1 \end{array}\right)$$

where

$$\delta_{\text{ij}}=\left\{\begin{array}{ll} 0, & \text{if}\;\;p_{i}=s_{j},\\ 1, & \text{otherwise}. \end{array}\right)$$

with initialization at the upper boundary by *C*[0,*j*]=0, and at the left boundary by *C*[*i*,0]=*i*, for *i*=1,2,…,*m* and *j*=1,2,…,*n*. Finally, the algorithm tests the last row of the matrix, i.e. *C*[*m*,*j*], and reports those elements that are no greater than *k*. This algorithm has *O*(*mn*) time and *O*(*mn*) space complexity.

The space bound can be easily reduced to *O*(*m*) if matrix *C* is computed by columns, noted as *C*_*j*_ for *j*=1,2,…*n*, and report a match each time *C*_*j*_[*m*]≤*k*, because computing column *C*_*j*_ requires only the knowledge of previous column *C*_*j*−1_. With careful design, *C*_*j*_ and *C*_*j*−1_ can share one column vector, as proposed by Ukkonen [4].

Ukkonen also observed that for columns that have the last element greater than *k*, there is a boundary index of *C*_*j*_, noted as *lac*(*C*_*j*_), such that *C*_*j*_[*lac*(*C*_*j*_)]=*k* and *C*_*j*_[*l*]>*k* for *l*=*lac*(*C*_*j*_)+1,…*m*. It is easy to prove that *lac*(*C*_*j*_)≤*lac*(*C*_*j*−1_)+1. Using this observation, Ukkonen reduced the time from *O*(*mn*) to expected *O*(*kn*) [4].

Our algorithm was developed from Ukkonen’s algorithm; however, we use a queue instead of an array to store all elements of current column above the boundary index. When there is a new element that corresponds to the topmost element of the new column, all elements in the queue shift automatically to the next (lower) position, just as elements transfer in the diagonals of matrix *C*. This process inherently keeps the basic properties of Ukkonen’s algorithm and facilitates subsequent improvements.

#### Lemma 1

In the dynamic programming matrix *C* for tackling the *k*-difference problem, the values of elements along each diagonal are monotonically non-decreasing.

The proof is provided in Additional file 5 Appendix A.

#### Theorem 1

All the matched elements of the query pattern and sequence are equal to their precursors in the diagonal and do not need to be updated in the dynamic programming process.

#### *Proof*.

This theorem is a direct consequence of Lemma 1 and the dynamic programming recurrence, when *δ*_*i* *j*_=0.

In other words, only mismatched elements need to be updated in the dynamic programming process. If bit-vectors that denote mismatched positions of comparison between the adapter sequence and each of the four nucleotide characters are pre-computed and a bit-vector that marks all positions of the queue elements that exceed the *k*-difference constraint is maintained, then unnecessary computations in updating the column vector can be inhibited. This is the key point that led to the main improvement of our algorithm over Ukkonen’s algorithm.

As listed in Algorithm 1, the *bit-masked k-difference matching algorithm* has the following characteristics: 

• Use a queue instead of an array to store all elements of the current column above boundary index.

• In preprocessing, calculate for each of four nucleotide characters a bit-vector that denotes the mismatched positions compared with the adapter.

• Mark the internal cells that exceed the *k*-difference constraint by a bit-vector which shifts as the queue pushes it.

• When processing the column starting from each input nucleotide, update only the cells that mismatch and have not been marked.

This algorithm uses a queue of size *m* and several bit vectors of size ⌈*m*/*w*⌉, where *w* is the word length of the computer (for example *w* equals 64 for a 64-bit machine), and hence has a space of *O*(*m*). For each of the *n* characters in a target sequence, the character enters the queue once and exits from the queue at most once. For a random sequence, the expected size of the queue is *O*(*k*); hence, generally the algorithm has *O*(*kn*) expected time. However, because it is restricted by the bit-mask operations, each element in the queue usually updates at most *k*+1 times. Because bit operations are negligible compared with element update operations, this algorithm achieves *O*(*kn*) worst-case time in practice, which is better than the *O*(*kn*) expected time for Ukkonen’s algorithm.

Algorithm 1 can be improved further by avoiding all unnecessary updates through constant time bit operations within each iteration cycle of the target nucleotide. The basic principle is that when an element in a diagonal of the original dynamic programming matrix has a value that is derived from an adjacent diagonal (i.e. an indel occurs in the corresponding path), the score and associated index of the element will remain unchanged if the precursor remains unchanged.

Although the above improvement can reduce the theoretical time complexity from expected *O*(*kn*) to worst-case *O*(*kn*), experiments on large volumes of real data showed that the reduced element update operations did not compensate for the additional bit operations.

For the *extended k-difference problem*, an additional step bounded by *O*(*m*) is performed to check all the elements remained in the queue if no hit was found in previous steps.

### Deal with base-call qualities

The main advantage of the *bit-masked k-difference matching algorithm* over *Myer’s bit-vector algorithm* is that it can be extended to handle base-call quality values.

To handle base-call quality values, we introduce the following parameters: *P*_*m* *i* *n*_=−*log*_10_(1/3), the minimum penalty for a mismatch; *P*_*m* *a* *x*_=−*log*_10_(10^40/(−10)^/3), the maximum penalty for a mismatch; *delta*=*P*_*m* *a* *x*_, the penalty for an insertion or deletion. The penalty of a mismatch with quality value *q* is calculated as:

$$\begin{array}{cccc} P(q)=\left\{\begin{array}{cc} P_{\text{min}} & q\leq 0\\ P_{\text{min}}+q/40\times (P_{\text{max}}-P_{\text{min}}) & 0<q<40\\ P_{\text{max}} & q\geq 40 \end{array}\right) & & & \end{array}$$

It is trivial to prove that *P*(*q*)=−*log*_10_(10^*q*/(−10)^/3) when 0<*q*<40. This score is the negative logarithm of the probability that the corresponding base is actually a match to the adapter sequence with sequencing error.

Note that the scoring scheme herein only induces penalties for mismatches and insertions/deletions. For matches, because the possibility of false matching based on sequence homology reduces exponentially as the matching length increases, the false matching possibility can be reduced by setting a longer alignment length. When PE information is available, the false matching possibility can be reduced to a minimum.

The extended version of Algorithm 1 that can take quality values into consideration is outlined in Additional file 6 Appendix B.

### Deal with paired-end information

**Figure 5:**
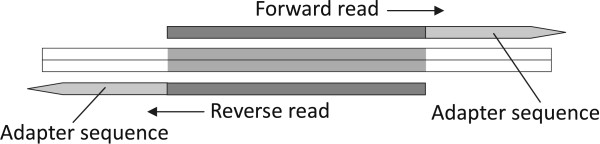
Layout of paired-end reads that have adapter contaminants.

Unlike the standard SE sequencing, PE sequencing reads out each DNA template twice, in opposite directions from different ends. The underlying fact is that all the PE reads that need to be trimmed must have the preserved paired sequences reverse-complement to each other, as illustrated in Figure 5 and Additional file 7 Appendix C.

Using this property, the program first finds all *k*-difference occurrences of adapters in both paired reads using the extended version of Algorithm 1 with quality values considered. Then the reverse-complementary property of each trimmed paired sequences is checked. Next, all candidates are evaluated with a scoring scheme that takes into account the fitness of adapter sequences in paired reads and the alignment of reverse-complementary counter-parts. Finally, the program outputs the optimal occurrence, if any.

The scoring scheme we used is as follows:

$$\begin{array}{ll} \;\text{score}(\text{idx})= & \text{pscore}(\text{read}1[\text{idx}\ldots \text{readLen}-1],\text{adapter}1)\\ & +\text{pscore}(\text{read}2[\text{idx}\ldots \text{readLen}-1],\text{adapter}2)\\ & +\text{pscore}(\text{read}1[0\ldots \text{idx}-1],\\ & \text{revComp}(\text{read}2[0\ldots \text{idx}-1])) \end{array}$$

where *idx* is the start position for trimming, *pscore*(*x*,*y*)=|*x*|×*P*_*m* *a* *x*_−*penalty*(*x*,*y*), *P*_*m* *a* *x*_ is the maximum penalty for a difference, *penalty*(*x*,*y*) is the penalty for matching *x* and *y* as calculated by the *k-difference matching algorithms*, and *revComp*(*x*) denotes the reverse complementary sequence of *x*. The goal is to find the *idx* that meets the *k*-difference requirement and maximizes the *score* function.

### Deal with Nextera LMP information

Mate-pair library sequencing allows the generation of long-insert PE libraries that are useful in the scaffolding process of de novo genome assembly and in the detection of long-range genome structural variations. In the Nextera LMP library construction process, there are additional reactions called “tagmentation” and “circularization” before the normal PE library construction. The tagmentation reaction uses a specially engineered transposome to fragment the DNA sample and tag the DNA fragments by attaching a pair of biotinylated junction adapters simultaneously to the ends. Next, the tagmented DNA molecules are circularized and sheared by ultrasonics, and the sub-fragments containing the original junction parts are enriched via the biotin tag in the junction adapter.

Trimming adapters from Nextera LMP reads is like a reverse process of Nextera LMP library construction. To process Nextera mate-pair reads, the program first trims the adapters as if it is dealing with PE reads. Then, it trims junction adapters from the processed paired reads separately using the extended version of Algorithm 1.

## Availability of supporting data

The data sets supporting the results of this article are available from high-throughput DNA and RNA sequence read archive: http://www.ncbi.nlm.nih.gov/sra/?term=SRR014966, http://www.ncbi.nlm.nih.gov/sra/?term=SRR330569, http:// www.ncbi.nlm.nih.gov/sra/?term=ERA264981, and http:// www.ncbi.nlm.nih.gov/sra/?term=SRR519624.

## Competing interests

The authors declare that they have no competing interests.

## Authors’ contributions

SZ and SWD supervised the project. HJ conceived the algorithm, developed the program, and performed the data analysis. RL explained NGS protocols and provided biological perspectives on trimming effects. All authors read and approved the final manuscript.

## Supplementary Material

Additional file 1 Tabular metrics of experiments on simulated data. Click here for file

Additional file 2 Tabular metrics of experiments on real sRNA data. Click here for file

Additional file 3 Tabular metrics of experiments on real PE RNA-Seq data. Click here for file

Additional file 4 Commands for conducting experiments on real LMP data. Click here for file

Additional file 5 Proofs of some fundamental lemmas. Click here for file

Additional file 6 Details of extended algorithms. Click here for file

Additional file 7 An example for adapter trimming of real PE data. Click here for file
