# Supplementary material for: Skewer: a fast and accurate adapter trimmer for next-generation sequencing paired-end reads
Source: BMC Bioinformatics. 2014 Jun 12;15:182. doi: 10.1186/1471-2105-15-182 (PMC4074385; doi:10.1186/1471-2105-15-182)
Supplement: Additional file 4 — Commands for conducting experiments on real LMP data. [file 1471-2105-15-182-S4.pdf]

Experiment on Nextera long mate pair (LMP) data for Arabidopsis Thaliana

1.a) command for trimming adapters from Nextera Long Mate Pair library [ENA:ERR365834] by NextClip:

```
$ time -p nextclip -i ERR365834_1.fastq -j ERR365834_2.fastq -o nextcliped
```

1.b) command for assembling paired-end library [ENA:SRR519624] and NextClip-trimmed Nextera Long Mate Pair library [ENA:ERR365834]:

```
$ abyss-pe k=39 name=athaliana lib='pe1' mp='mp1 mp2 mp3' pe1='
SRR519624_1.fastq SRR519624_2.fastq' mp1='nextcliped_A_R1.fastq
nextcliped_A_R2.fastq' mp2='nextcliped_B_R1.fastq nextcliped_B_R2.fastq'
mp3='nextcliped_C_R1.fastq nextcliped_C_R2.fastq'
```

2.a) commands for trimming adapters from Nextera Long Mate Pair library [ENA:ERR365834] by skewer:

```
$ time -p skewer -m mp ERR365834_1.fastq ERR365834_2.fastq -t 8 -o
ERR365834-trimmed
```

2.b) command for assembling paired-end library [ENA:SRR519624] and skewer-trimmed Nextera Long Mate Pair library [ENA:ERR365834]:

```
$ abyss-pe k=39 name=athaliana lib='pe1' mp='mp1' pe1='SRR519624_1.fastq
SRR519624_2.fastq' mp1='ERR365834-trimmed-pair1.fastq
ERR365834-trimmed-pair2.fastq'
```

References:

[1] Leggett RM, Clavijo BJ, Clissold L, Clark MD, Caccamo M: **NextClip: an analysis and read preparation tool for Nextera Long Mate Pair libraries**. *Bioinformatics* 2014, **30**(4):566-568.

[2] Jiang, H. **Skewer** (v0.1.114). http://sourceforge.net/projects/skewer/, 2013

[3] Simpson JT, Wong K, Jackman SD, Schein JE, Jones SJ, Birol I: **ABySS: a parallel assembler for short read sequence data**. *Genome Res* 2009, **19**(6):1117-1123.
