# Supplementary material for: Skewer: a fast and accurate adapter trimmer for next-generation sequencing paired-end reads
Source: BMC Bioinformatics. 2014 Jun 12;15:182. doi: 10.1186/1471-2105-15-182 (PMC4074385; doi:10.1186/1471-2105-15-182)
Supplement: Additional file 5 — Proofs of some fundamental lemmas. [file 1471-2105-15-182-S5.pdf]

Appendix A. Proofs

Lemma 1. In the Dynamic Programming Matrix $C_{m\times n}$ for tackling the *k-difference Problem*, the values of elements along each diagonal are monotonically non-decreasing.

Proof: For any two adjacent elements along a diagonal, if the data flow to the right bottom element is from the left top one, then as the penalty (or distance) is non-negative and accumulative, the right bottom element cannot be smaller than the left top one; otherwise, without loss of generality, suppose the data flow to the right bottom element[1] $C_{i,j}$ is from its top neighbor $C_{i-1,j}$, and suppose $C_{i,j}<C_{i-1,j-1}$, then $C_{i-1,j}=C_{i,j}-1<C_{i-1,j-1}-1$, so the data flow to $C_{i-1,j}$ are from neither $C_{i-1,j-1}$ nor $C_{i-2,j-1}$, otherwise result in $C_{i-1,j-1}<C_{i-1,j}<C_{i-1,j-1}-1$ or $C_{i-2,j-1}\leq C_{i-1,j}<C_{i-1,j-1}-1$, but from the recurrence logic, $C_{i-2,j-1}\geq C_{i-1,j-1}-1$, contradictory. The same reasoning is applied for all the elements above $C_{i,j}$, and finally we have $C_{0,j}=C_{i,j}-i<C_{i-1,j-1}-i$. But from the recurrence logic, we have $C_{0,j-1}\geq C_{1,j}-1\geq\ldots\geq C_{i-1,j-1}-(i-1)$, so $C_{0,j-1}-C_{0,j}>1$. Which is not true for the recurrence start condition. In conclusion, in all cases $C_{i,j}\geq C_{i-1,j-1}$. ■

Lemma 2. If the penalty for an insertion/deletion is D, the maximum penalty for a mismatch is P, then $\Delta h_{i,j}=C_{i,j}-C_{i,j-1}\in[-D,D]$; $\Delta v_{i,j}=C_{i,j}-C_{i-1,j}\in[-D,D]$; $\Delta d_{i,j}=C_{i,j}-C_{i-1,j-1}\in[0,\min(P,2D)]$.

Proof: From the dynamic programming recurrence, $C_{i,j}\leq C_{i,j-1}+D$, thus $C_{i,j}-C_{i,j-1}\leq D$. From Lemma 1, $C_{i,j}\geq C_{i-1,j-1}\geq C_{i,j-1}-D$, thus $C_{i,j}-C_{i,j-1}\geq -D$, hence, $C_{i,j}-C_{i,j-1}\in[-D,D]$; similarly, $C_{i,j}-C_{i-1,j}\in[-D,D]$; From Lemma 1, $C_{i,j}-C_{i-1,j-1}\geq 0$, From the dynamic programming recurrence $C_{i,j}-C_{i-1,j-1}\leq P$; also $C_{i,j}\leq C_{i-1,j}+D\leq C_{i-1,j-1}+2D$, thus $C_{i,j}-C_{i-1,j-1}\in[0,\min(P,2D)]$. ■

---

[1] $C_{i,j}$ and C[i,j] denote the same element in our nomenclature
