# Supplementary material for: Skewer: a fast and accurate adapter trimmer for next-generation sequencing paired-end reads
Source: BMC Bioinformatics. 2014 Jun 12;15:182. doi: 10.1186/1471-2105-15-182 (PMC4074385; doi:10.1186/1471-2105-15-182)
Supplement: Additional file 6 — Details of extended algorithms. [file 1471-2105-15-182-S6.pdf]

## Appendix B. Algorithms

**Algorithm 2.** *bit-masked k-differences matching algorithm with quality values aware*[1]

```
Preprocessing
for a in [A,C,G,T,N] do
   calculate a bit vector misBits[a] in comparison with P
end for
Alignment
legalBits = 0
penalK = k × ((P_min + P_max) / 2)
for i=1 to ⌊penalK/delta⌋ do
   Q.pushBack(idx=-i,dif=delta×i)
   legalBits = (legalBits << 1) | 1
end for
for j=0 to n-1 do
   d = (P_0≠S_j)? qual2penalty(qv_j):0
   Q.pushFront(idx=j,dif=d)
   legalBits = (legalBits << 1) | 1
   for i=1 to Q.size()-1 && (legalBits(i) & misBits[S_j](i)) do
      Q[i].dif = Q[i].dif + (qual2penalty(qv_j) - delta)
      q=argmin_{r=i-1}^{i+1}(Q[r].dif)
      {r≠i+1 for the last iteration}
      Q[i].dif = Q[q].dif + delta
      Q[i].idx = Q[q].idx
      if Q[i].dif > penalK then
         legalBits &= ~(1 << i)
      end if
   end for
   while Q.back().dif > penalK do
      Q.popBack()
   end while
   if Q.size() == m then
      report Q.back().idx;
      Q.popBack()
   end if
end for
function qual2penalty(qual)
   return P_min + qual/quality_max × (P_max - P_min)
end
```

[1] $P_{min}$: minimum penalty for a mismatch; $P_{max}$: maximum penalty for a mismatch; delta=$P_{max}$: penalty for an indel; $quality_{max}$=40: maximum possible quality value; *qv*: quality sequence corresponding to *S*
