# Supplementary material for: Skewer: a fast and accurate adapter trimmer for next-generation sequencing paired-end reads
Source: BMC Bioinformatics. 2014 Jun 12;15:182. doi: 10.1186/1471-2105-15-182 (PMC4074385; doi:10.1186/1471-2105-15-182)
Supplement: Additional file 7 — An example for adapter trimming of real PE data. [file 1471-2105-15-182-S7.pdf]

Appendix C. An example of trimming real paired-end reads

>paired-read1
AGCTGACGTAATACCTATTTGTGGCATAACAAGAAAAGGGGATAGATATATGTAGAGAAAAGATCG
GNNGAGCACACGTCTGAACTCCAGTCACACANTGA
>paired-read2
TTTCTCTNNNNNNNNNNNNCCCCTTTTCTTGTTATGCCACAAATAGGTATTACGTCANNNNNNNNN
NNNNNNNNNNNNNNNNNNNNNNNNNNNNNNNNNNN

>trimmed
AGCTGACGTAATACCTATTTGTGGCATAACAAGAAAAGGGGATAGATATATGTAGAGAAA
